# Bacteria-laden microgels as autonomous three-dimensional environments for stem cell engineering

**DOI:** 10.1016/j.mtbio.2019.100011

**Published:** 2019-06-18

**Authors:** K. Witte, A. Rodrigo-Navarro, M. Salmeron-Sanchez

**Affiliations:** Center for the Cellular Microenvironment, University of Glasgow, G12 8LT, UK

**Keywords:** Droplet-based microfluidics, Bioprinting, Engineered bacteria, Cell engineering, Living materials, Stem cells

## Abstract

A one-step microfluidic system is developed in this study which enables the encapsulation of stem cells and genetically engineered non-pathogenic bacteria into a so-called three-dimensional (3D) pearl lace–like microgel of alginate with high level of monodispersity and cell viability. The alginate-based microgel constitutes living materials that control stem cell differentiation in either an autonomous or heteronomous manner. The bacteria (*Lactococcus lactis*) encapsulated within the construct surface display adhesion fragments (III_7-10_ fragment of human fibronectin) for integrin binding while secreting growth factors (recombinant human bone morphogenetic protein-2) to induce osteogenic differentiation of human bone marrow–derived mesenchymal stem cells. We concentrate on interlinked pearl lace microgels that enabled us to prototype a low-cost 3D bioprinting platform with highly tunable properties.

## 1 Introduction

Cells in tissues of multicellular organisms are enclosed in networks made of proteins and polysaccharides called the extracellular matrix (ECM). In addition to the physical support that it provides for tissues, the ECM initiates crucial biochemical and biomechanical cues required for the survival, development, migration, proliferation, shape, and function of cells that are embedded. It is a dynamic network which often gets remodeled by the cells inhabiting it [1].

Hydrogels as synthetic matrices have received considerable interest for biomaterial applications owning to their compositional and structural similarities to the natural ECM [2]. They make three-dimensional (3D) networks composed of hydrophilic polymers cross-linked either through covalent bonds or held together via physical intramolecular and intermolecular interactions [3], [4]. Alginate has been one of the more extensively investigated and used hydrogels in biomedical applications. It is a naturally occurring anionic polymer with a high biocompatibility, low cytotoxicity, relatively low cost, and mild cross-linking by addition of divalent cations such as calcium [5]. A major challenge in tissue engineering is the diffusion limit of 100–200 ​μm for oxygen as well as nutrients and essential biomolecules to the cell population embedded in the hydrogels [2], [6]. One way to overcome the diffusion limitation, besides vascularization [7], is to assemble small (sub-mm), cell-containing modules to form larger tissue constructs with homogeneous material properties [8], [9]. Most cell microencapsulation techniques are based on droplet extrusion methods, where cells are mixed with the alginate solution and then extruded through a needle, forming droplets of varying sizes and population densities which are collected in a gelling medium usually containing Ca^2+^ [6].

The use of microfluidics, however, allows for the formation of monodisperse microcapsules [10], [11] or fibrous constructs [12], [13] with fine control over cell population density and coculture of cell populations in well-defined mixing ratio. The rapid ion exchange between calcium and alginate results in a fast gel formation, and current microfluidic methods to produce microcapsules are carried out in complex multistep processes [10], [11]. In case of fibrous constructs, reported fabrication methods involve extruding long thread-like structures made of alginate while keeping it sheathed and polymerized by a secondary aqueous solution carrying other biopolymers such as gelatine [14] and sucrose [15] together with Ca^2+^. In contrast, we developed a one-step droplet microfluidic method for fabrication of cell-laden pearl lace microgels made from alginate which is performed at physiological pH without using sheathing material to preserve cell viability. The level of fluid shear stress experienced by cells as they travel through the capillary channel can influence their viability and mechanosensitivity*.* For instance, mechanically induced activation of signaling pathways by shear stress affects cell behavior [16] or their plasticity in case of stem cells [17], [18]. We addressed this issue in our microfluidic device by choosing appropriate channel dimensions and designs in conjunction with reduced flow rates.

The fabricated gel construct is unique in a way that it has both compartmentalized units as in individual microcapsules as well as the connectivity found in fibrous constructs. It is also noted that the compartmentalized microunits and the link connecting them are highly tunable resulting in highly monodispersed pearl lace interlinking structures. Inherent to the fabrication process, the pearl unit compared to the interlinking unit benefit from a slower cross-linking in addition to a dampened shear stress*.* This technology allows manufacturing compartmentalized yet linked cell-laden hydrogels with unprecedented precision and control [19], [20], [21] which has been exploited here as a low-cost 3D bioprinting prototype.

As a proof of concept, we have used this microfluidic technology to automate fabrication of an in vitro 3D model for investigating the commensalism symbiosis between eukaryotic (human bone marrow–derived mesenchymal stem cells ​[hBM-MSCs]) and prokaryotic cells (engineered non-pathogenic bacteria *Lactococcus lactis*).

Bacteria are long being utilized as a fast and low-cost production organism of proteins and glycoproteins of biomedical interest [[22], [23], [24], [25], [26]]. As it has been demonstrated by Hay et al., they can also be used to provide a spatiotemporal requested proteins and factors to direct cell proliferation and differentiation. For instance, bone morphogenetic protein-2 (BMP-2), a well-known growth factor for inducing new bone formation [27], has been engineered to be released in a controllable fashion by *L. lactis* to create a dynamic functional living biointerphases between synthetic materials and living cells [28], [29]. In addition to release of BMP-2 (protein of interest), *L. lactis*'s surface was engineered to accommodate as the binding site for integrins by incorporating arginine-glycine-arpartic acid (RGD)-containing motif of fibronectin (FN). FN ​mediates a wide variety of cellular interactions with the ECM ​and plays important roles in cell adhesion, migration, growth, and differentiation [27], [30], [31]. Because MSCs do not have receptors that recognize alginate, in this study, the combination of these two proteins and their synergistic effect on differentiation for modeling a micron-sized 3D model of osteogenesis were explored.

The homofermentative metabolism of *L. lactis* in presence of a suitable carbon source leads to a buildup of lactic acid, therefore acidifying the media which is detrimental to MSC ​viability. To prevent acidification due to the accumulation of lactic acid within the microparticles, a lactate dehydrogenase (LDH)–deficient strain of *L. lactis*, NZ9020, was used [32]. LDH, which metabolizes pyruvate to lactic acid, is the last enzyme in the homofermentative glycolytic (Embden–Meyerhof–Parnas) pathway characteristic of the lactic acid bacteria clade [32]. To prevent bacterial proliferation inside the microgels, sulfamethoxazole (SMX), a bacteriostatic antibiotic that prevents folic acid synthesis, was used. Note that mammalian cells, unable to synthesize folic acid, are insensitive to this antibiotic [33]. In one study, it has been reported that SMX in combination with trimethoprim antibiotic reduces peripheral blood stem cell ​mobilization while not affecting the quantity of the cell assessed [34]. In studies by Hay et al. [28], the low concentration of SMX alone concluded no adverse effect on MSC ​viability and its osteogenic differentiation; however, the mobility of the cells was not investigated.

## 2 Material and methods

### 2.1 *L. lactis* cell culture

Two transformants of *L. lactis* (ldhA^−^ and ldhB^−^) were engineered [28]. One of the transformants contained a constitutive expression vector carrying the gene for FNIII_7-10_ fragment. The other one contained a constitutive expression vector carrying the gene for BMP-2. The two vectors had chloramphenicol resistance gene. Bacteria were cultured at 30 ​°C in anaerobic conditions, in M17 medium (Oxoid, Basingstoke, UK) supplemented with glucose (0.5% v/v, Sigma), erythromycin (10 μg/mL, Sigma-Aldrich), and chloramphenicol (10 μg/mL, Sigma-Aldrich). Same gene constructs cloned in nisin-inducible plasmids containing chloramphenicol resistance gene were used for inducible studies. Daily change of media with 10 ​ng/mL nisin was used for inducing protein expression of BMP-2 and FNIII_7-10._

### 2.2 Human MSC culture

Dulbecco's Modified Eagle Medium (DMEM) (PromoCell GmbH) supplemented with 4.5 ​g/L glucose, 100 ​μM sodium pyruvate, 1 ​mM l-glutamine, 10% fetal bovine serum (FBS), and 100 U/mL penicillin/streptomycin was used for maintaining hBM-MSCs (Promocell). Cultures were kept at 37 ​°C and 5% CO_2_ in a humidified atmosphere. Media was changed every 2–3 days. For experimental use, MSCs were maintained between passages p2 and 4.

### 2.3 *L. lactis* and MSC coculture

MSC media was changed to DMEM supplemented with 2% FBS, 1 ​g/L glucose, 100 ​μM sodium pyruvate, 1 ​mM l-glutamine, and 10 μ g/mL chloramphenicol, and in order to inhibit bacterial metabolism and control media acidification, 10 ​μg/mL SMX and 10 ​μg/mL tetracycline were added. Cultures were kept at 37 ​°C and 5% CO2 in a humidified atmosphere. Encapsulated cells were cultured for two weeks with media being changed every day. Osteogenic media was prepared by supplementing DMEM with 2% FBS, 1 ​g/L glucose, 100 ​μM sodium pyruvate, 1 ​mM l-glutamine, 25 ​μg/ml l-ascorbic acid, 0.1 ​μM dexamethasone, 3 ​mM NaH2PO4, and 100 U/mL P/S. Encapsulated MSCs were cultured for two weeks with media being changed every 2–3 days.

### 2.4 Microfluidic fabrication

**Fig. 1:**
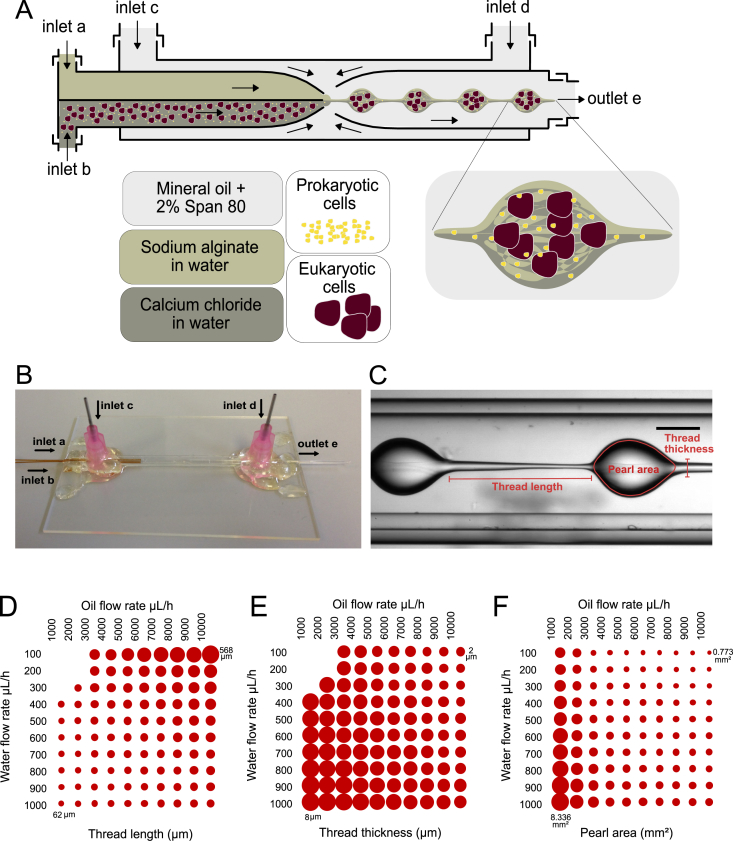
**Droplet-based microfluidic setup.** (A) Schematic representation of the microfluidic device and encapsulation of prokaryotic and eukaryotic cells. (B) Image of the capillary-based microfluidic device. (C) A snapshot of pearl formation in microfluidic device with indication of parameters used to quantify assembled pearls. (D) Thread thickness graph with corresponding flow rates (Y-axis: water flow; X-axis: oil flow). (E) Thread thickness graph. (F) Pearl area graph.

Septum theta (TST150-6) with 1.5/1.02 outer diameter (OD)/inner diameter (ID) (mm) and thin wall round glass capillaries (TW100-6) with 1/0.75 OD/ID (mm), purchased from World Precision Instruments, Inc., were tapered (0.4 ​mm diameter orifice) using a Sutter Instruments model P-97 micropipette puller. Larger diameters of tapered tips were made using sand paper. Smaller sizes of tapered tips were made manually by pulling locally heated capillaries. Square capillary of 2 ​mm ​± ​6% ID with wall thickness of 0.4 ​mm was purchased from World Precision Instruments, Inc. Capillary surfaces were modified with hydrophobic Sigmacote (Sigma-Aldrich). Silica capillaries were first cleaned by soaking in a piranha solution (3:1 sulfuric acid:hydrogen peroxide) for 20 ​min, followed by rinsing with deionized water and drying with nitrogen gas. The treated capillaries were rinsed with isopropanol and dried with nitrogen gas. The device was fabricated by coaxially aligning two round capillaries with tapered ends facing each other inside a square capillary as shown in Fig. 1A. One of the two round capillaries had a theta ϴ-shaped cross section with OD of 2.00 ​± ​0.10 ​mm and ID of 1.40 ​± ​0.10 ​mm. The other round capillary is hollow with OD of 1.80 ​mm and ID of 1.50 ​mm. The square capillary inner width is 2.00 ​mm with wall thickness of 0.40 ​mm. Each of the two theta-shaped capillary channels was connected to polytetrafluoroethylene (PTFE) soft tubing via small capillaries inserted from the untapered end for introducing aqueous solutions into the device in a synchronous manner (Fig. 1B). The microfluidic device was coupled with controllable syringe pumps (Harvard Apparatus) for fluid delivery.

### 2.5 Cell encapsulation

*L. lactis* and MSCs were individually resuspended in DMEM supplemented with 2% FBS, 1 ​g/L glucose, 100 ​μM sodium pyruvate, 1 ​mM l-glutamine, and 10 μ g/mL chloramphenicol. Cells were then pooled and mixed 1:1 with solution containing 4% (w/v) medium viscosity sodium alginate (Sigma-Aldrich, UK) in order to make a 2% alginate solution. Cell mixture with alginic acid was introduced into the device via one of the two channels of theta-shaped capillaries and 200 ​mM calcium chloride solution from the other channel. Mineral oil (Sigma-Aldrich) with 2% Span 80 (Sigma-Aldrich) was pumped through a square capillary in order to form the continuous phase. Dispersed hydrogel droplets were collected in a prewarmed culture media, followed by washing with 100 ​mM calcium chloride and culturing in DMEM media supplemented with 2% FBS, 1 ​g/L glucose, 100 ​μM sodium pyruvate, 1 ​mM l-glutamine, 10 ​μg/mL chloramphenicol, 10 ​μg/mL SMX, and 10 ​μg/mL tetracycline at 37 ​°C and 5% CO_2_ in a humidified atmosphere. Media was changed every day for gels containing bacteria and every 2–3 days for gels containing MSCs only.

### 2.6 Viability assay

For bacterial viability studies, alginate constructs embedded with cells were washed twice with 100 ​mM calcium chloride solution prior to incubation with 5 ​μM SYTO^®^ 9 and 30 ​μM Propidium iodide (BacLight LIVE/DEAD kit, Life Technologies, UK) in 100 ​mM calcium chloride solution for an hour at 37 ​°C. For mammalian cell viability studies, encapsulated cells in hydrogel were incubated in a 100 ​mM calcium chloride solution containing 2 ​μM calcein and 2 ​μM ethidium homodimer-1 (Viability/Cytotoxicity Kit, Life Technologies) for an hour at 37 ​°C. Viability was calculated by analyzing the total amount of cells stained in green versus the number of cells stained in red and green. The distribution of live cells (green) and dead cells (red) was visualized using EVOS Fl Colour Cell Imaging System and software. Acquired images were analyzed by quantifying pixel intensities of the fluorescence signals using the ImageJ software version 1.51 (100) and plotted using GraphPad Prism (version 7.0 software). Merged fluorescence channel images were processed using the same version of the ImageJ software.

### 2.7 Immunofluorescence

Hydrogel constructs containing cells were washed twice in 100 ​mM calcium chloride before getting fixed in 4% (w/v) paraformaldehyde for 30 ​min at room temperature. Samples were permeabilised in 0.01% (v/v) Triton X-100 for 30 ​min and blocked with 1% bovine serum albumin (BSA) in 100 ​mM calcium chloride for an hour at room temperature. Samples were then incubated overnight with the following primary antibodies: osteocalcin (OCN, mouse monoclonal, sc-365797, UK) or osteopontin (OPN, mouse monoclonal, sc-21742, UK) (Santa Cruz Biotechnology, UK) diluted 1:50 in 100 ​mM calcium chloride, 1% BSA. On the following day, samples were washed three times with 0.5% Tween 20 in 100 ​mM calcium chloride for 5 ​min each wash before incubation with a secondary, biotin-conjugated antibody (monoclonal rabbit anti-mouse [IgG], Vector Laboratories, UK) diluted 1:50 in 100 ​mM calcium chloride, 1% BSA. Dilution of 1:50 Alexa Fluor 488-conjugated phalloidin (Life Technologies) was added to the samples for the duration of this incubation. Samples were then washed three times with 0.5% Tween 20 in 100 ​mM calcium chloride for 5 ​min each wash. Samples were incubated with 1:50 dilution of rhodamine-conjugated streptavidin (Vector Laboratories) and 0.03 ​mM PureBlu Nuclear Staining (Bio-Rad, UK) in 100 ​mM calcium chloride, 1% BSA for 30 ​min at 4 ​°C. Hydrogels were then washed three times with 0.5% Tween 20 in 100 ​mM calcium chloride for 5 ​min each wash prior to fluorescence imaging using a Zeiss AxioObserver- Z1 microscope or EVOS Fl Colour Cell Imaging System and software. Acquired images were analyzed with ImageJ as described previously.

### 2.8 Scanning electron microscopy

Hydrogel constructs containing cells were washed twice in 100 ​mM calcium chloride before getting fixed in 4% (w/v) paraformaldehyde for an hour at room temperature. Fixed hydrogel samples were washed three times for 5 ​min each wash with 0.15 ​M sodium cacodylate and incubated in 1% osmium tetroxide, 0.15 ​M sodium cacodylate buffer for an hour at room temperature. Samples were then washed five times in distilled water before dehydration through an ethanol series beginning with 30% and changing to solutions of 50%, 70%, 90%, and three times 100%, over 30 ​min. Samples were then dried with a Polaron E3000 Critical Point Dryer (supercritical CO_2_) for 80 ​min. Prior to imaging, samples were sputter coated with 1–2 ​nm gold-palladium using a POLARON SC515 SEM Coater. Samples were imaged in a JEOL 6400 SEM with a gun voltage acceleration of 10 ​kV.

### 2.9 Transmission electron microscopy

Similar to scanning electron microscopy, samples were dehydrated with ethanol solutions of 50%, 70%, 90%, and three times 100%, over 30 ​min prior to be washed with propylene oxide three times each for duration of 5 ​min. They were then mixed in 1:1 propylene oxide: Epon 812 resin overnight as propylene oxide evaporates. Samples were changed to fresh resin, embedded in flat bed molds and polymerized for 48 ​h at 60 ​°C. 60–70 ​nm ultrathin sections were produced using Leica Ultracut UCT and a Diatome diamond knife at an angle of 6 ​°C. Sample sections were picked up on 100 mesh formvar coated copper grids then contrast stained with 2% methanolic uranyl acetate for 5 ​min and Reynolds lead citrate for another 5 ​min. Samples were viewed on JEOL 1200 TEM at an accelerating voltage of 80 ​kV. TIF images were captured using Olympus iTEM soft imaging system.

### 2.10 3D printing

Four simple geometrical shapes (line, triangle, square, and circle) were made from poly(methyl methacrylate) (PMMA) using HPC laser cutter. The 3 ​mm in diameter circle discs were laser cut through 3.00 ​mm acrylic sheet and glued using epoxy on a second 3.0 ​mm acrylic sheet. The arrangement of discs for making each of the four shapes was as follow: 2 circular discs for line (180-degree angle); 3 for triangle (60-degree internal angle); 4 for square (90-degree internal angle), and 8 (135-degree internal angle, octagon) for a circle-like shape. One-micron-sized green (450/480) fluorescent polystyrene microspheres (Thermo Scientific™ Fluoro-Max) were encapsulated within alginate pearl lace construct. While the construct being continuously fabricated and collected from the microfluidic device, it was getting perpendicularly wrapped around the pillars (circular discs) after going through an aqueous solution (100 ​mM calcium chloride) and leaving behind mineral oil. Between two to four turns were made around each pillar. The bright-field and fluorescent images were taken by scanning over a 30 ​× ​30 mm square using EVOS FL Colour Cell Imaging System at 10X objective and stitched together using EVOS owns software. The hydrogels were air-dried prior to imaging.

### 2.11 Statistical analysis

Data were presented as mean values, standard deviations, and/or standard error. Mean values were calculated from two independent experiments of triplicates by group. Statistical analysis was performed by one-way analysis of variance followed by a Tukey post hoc test in GraphPad Prism™ 6 software using a level of significance of p ​< ​0.1 (*), p ​< ​0.05 (**), p ​< ​0.001 (***), and p ​< ​0.0001 (****).

## 3 Results and discussion

### 3.1 Microfluidic fabrication of monodispersed alginate microgels

The production of alginate microgels was carried out via single-step emulsification in 3D (axisymmetric) flow focusing glass capillary microfluidic devices. The device design is based on a previous design by Lee et al. [11]; however, and importantly, the formation of the alginate construct in this report is fundamentally different and is performed within a single step as oppose to their reported three-step formation of microgels. Each of the two channels of theta ϴ-shaped capillaries carries aqueous solutions, one carries 2% (w/v) sodium alginate and the other one carries 100 ​mM calcium chloride. At the tapered end, the two aqueous fluids meet and form microgels while simultaneously getting dispersed in mineral oil with 2% Span 80 pumped through via the square capillary. The hollow round capillary at the other end of the device is the outlet for the formed hydrogel construct and its carrier oil. Microgels then collected in 100 ​mM calcium chloride solution as the hydrogel sink and the less-dense oil (density of 0.84 ​g ​mL^−1^) and surfactant float ​on top of the solution.

**Fig. 2:**
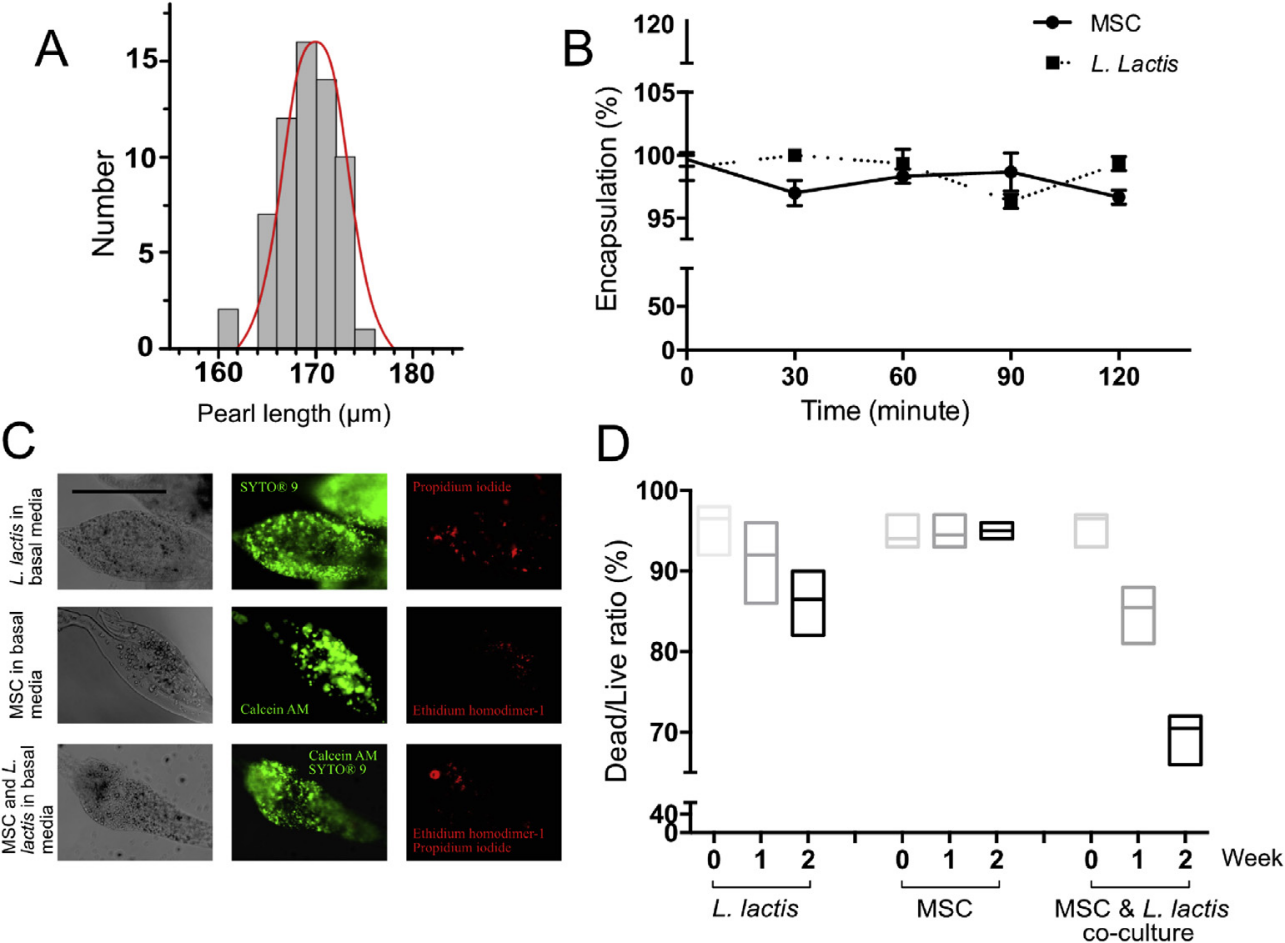
**Monodispersity, encapsulation, and viability of microbeads.** (A) Pearl size (long axis) distribution of produced hydrogel of two miscible fluid streams under laminar flow conditions using flow rates of 500 ​μL ​h^−1^ for the two inner phases and 5000 ​μL ​h^−1^ for the outer phase. The mean length of the formed pearl was 167 ​μm with an RSD of 3.2%. Scale bar: 100 ​μm. (B) Cell (MSCs and *L. lactis*) encapsulation efficiency. The cell counts at each time point are the result of 8 measurements sequentially acquired at 30-min intervals at room temperature for 2 ​h. (C) Fluorescent images of two-week-old alginate hydrogels with *L. lactis* and MSCs. The hydrogel was stained with *Bac*Light Bacterial Viability Kit for L. *lactis* and Viability/Cytotoxicity Kit, for MSCs. Both kits stain viable cells in green (SYTO^®^ 9 and Calcein AM) and non-viable cells in red (Propidium iodide and Ethidium homodimer-1), a 50:50 mixture of the kits were used for coculture. N > 5-10 microgels were analyzed for each condition. Scale bar: 100 ​μm. (D) The viability graph of *L. lactis,* MSCs, and coculture. MSCs, mesenchymal stem cells.

Different sizes of microgels were made by changing the flow rate of oil and water phase as shown in Fig. 1C–F. But once a set of flow rates were selected, the formed microgels were monodispersed in shape and size (Fig. 2A). Two distinctive microgels namely unlinked and linked pearl-shaped beads were made by altering the flow rates (Figs. S1(A and B) and Supplementary Videos 1 and 2).

Supplementary video related to this article can be found at https://doi.org/10.1016/j.mtbio.2019.100011

The following are the supplementary data related to this article:

Supplementary video 1 1 Supplementary video 1

Supplementary video 2 2 Supplementary video 2

Individual beads were made at relatively low flow rates (100–200 ​μL ​h^−1^ for water phase and 1000–2000 ​μL ​h^−1^ for oil phase) as compared to those of linked pearl beads (300–1000 ​μL ​h^−1^ for water and 3000–10000 ​μL ​h^−1^ for oil). The physical characteristics of linked pearl beads such as size, the distance, and the thickness of the link between two pearl beads can be fine-tuned by controlling the ratio between water to oil flow rates. For example, by increasing the rate of the oil phase, the thread linking two pearls can get extended (Fig. 1D). The same outcome can also be achieved by reducing the rate of the water phase. In a similar way, by increasing the flow rate of the oil phase, the thread thickness (Fig. 1E) and the pearl area (Fig. 1F) decrease in size, which can also be achieved by reducing the flow rate of the water phase. Furthermore, by reducing the rate of both water and oil phases, the link between pearls will not form, hence resulting in unlinked beads. The production rate of the hydrogels ranges from 10 to 100 units per second based on selected flow rates.

### 3.2 Encapsulation and viability of bacteria and MSCs in micron-sized hydrogels

Microfluidics provides a high throughput technology for encapsulation of different cell types as compared to more conventional and laborious methods, which provide low yield. A similar construct to pearl lace microgel has reported using in-air microfluidics [35]. However, owing to the fabrication nature of in-air microfluidic platform, constructs were cross-linked well after formation in a calcium chloride bathing solution, which resulted in microgel coalescence. It has also been noted that monodispersity and tunability of their constructs are limited compared to our droplet-based microfluidic platform in which liquid dispersion and cross-linking all happens simultaneously.

As the two aqueous solution one carrying alginate and one carrying Ca^2+^ meet at the tapered end of injection theta capillary, they simultaneously get focused within the laminar flow of mineral oil moving towards collection outlet. Cross-linking happens as Ca^2+^ diffuses through sodium alginate solution while microconstructs are formed under laminar flow using a coflow focusing method. This dynamic system results in the formation of monodisperse but not symmetrical spherical beads and pearls (Supplementary Fig. S1). It is important to point out that alginate-calcium hydrogel as well as many other crossed linked hydrogels are formed through diffusion of participating molecules. Because of the time-dependent nature of diffusion, the homogeneity of any formed construct can never be fully achieved. However, the smaller the construct the smaller the difference would be across the formed hydrogel. Also, in regard to cell distribution within the construct, the smaller the unit, on average, the more homogeneous would be the spread of the cells across it (see Supplementary Video 4).

The encapsulation is only achieved if cells are introduced via alginate solution as small Ca^2+^ molecules diffuse across to large molecules of alginate and not the other way around. Because ionic cross-linking occurs extremely rapidly (ms), cells introduced via aqueous solution containing calcium chloride would not be able to get entrapped in alginate network. As a consequence, they dispersed into aqueous collection solution together with excess water once no longer focused by mineral oil within glass boundary of the collection capillary.

We used *L. lactis* as our prokaryotic and BM-MSCs ​as our eukaryotic cell of interest. Initially, only one cell type was encapsulated in the hydrogel for studying the viability and encapsulation efficiency (Supplementary video of cell encapsulation, video 3). Either cell type once introduced via the channel carrying the alginate solution was encapsulated with well over 99% efficiency as shown in Fig. 2B. The measurement was made over the course of 2 ​h by staining the collection media for identifying cells that were not encapsulated.

The doubling time of wild-type *L. lactis* in cultures grown aerobically is 32–34 ​min. Its fast proliferation rate in hydrogel as shown in Fig. S1(C) was observed over the time course of three days. In the follow-up experiments in order to keep the cell density of the bacteria at a stationary phase, a combination of antibiotics (tetracycline and SMX) as well as a lower metabolically active strain of *L. lactis* deficient in LDH ​were used [32].

The volume of the pearl lace microgel is well defined, and hence, the space is limited for a fast dividing *L. lactis*, with an initial concentration of 1 million cells per mL of media the *L. lactis's* viability was kept at approximately 80% by two weeks of culturing (Fig. 2C–D, *L. lactis* in basal media). With MSCs ​the viability over the same time period was monitored to be well over 90% (Fig. 2C–D, MSCs in basal media). Because the viability of MSCs was as high as week zero, we kept some of the samples for longer (over four weeks) with no observed decreased in viability.

The most commonly used methods for forming coculture spheroids as 3D in vitro models such as hanging drop [36], soft agar liquid overlay [37], and functionalised microparticles [38] do not form monodisperse constructs in terms of size, population density, and cell type ratio. As shown in the supplementary videos (Supplementary Videos 3 and 4), we have exploited microfluidic technology for fabrication of monodispersed microgels in which the population of different cell types as well as their ratio and distribution within the gels can be finely controlled.

Supplementary video related to this article can be found at https://doi.org/10.1016/j.mtbio.2019.100011

The following is the supplementary data related to this article:

Supplementary video 3 3 Supplementary video 3

Supplementary video 4 Supplementary video 4

For coculture studies, we used two constructs for *L. lactis* previously reported by us [28]. The first of the two plasmids encodes the gene for an FN fragment containing RGD integrin-binding motifs (FNIII 7-10) displayed as membrane protein. The second plasmid encodes the human BMP-2 ​released into the extracellular medium by the bacteria. Both constitutive and inducible, i.e. triggered by external stimuli (nisin), versions of engineered genes were used for the following studies. Using the microfluidic approach, three cell populations (MSCs, FNIII 7-10 displaying *L. lactis*, and BMP-2 releasing *L. lactis*) were encapsulated into microgels after getting mixing in the channel carrying alginate. The fabricated microgels were incubated in culture medium at 37 ​°C, 5% CO_2_. The media was changed daily for two weeks. Microgels containing MSC-only cells encapsulated in a similar way using either basal media (negative control) or osteogenic media (positive control) were kept under the same incubation conditions. Samples were taken at day zero, seven, and fourteen. In single-cell studies, hydrogels were stained with *Bac*Light Bacterial Viability Kit for *L. lactis* and Viability/Cytotoxicity Kit, for MSCs. Both kits stain viable cells in green (SYTO^®^ 9 and Calcein AM) and non-viable cells in red (Propidium iodide and Ethidium homodimer-1). Note that either kit stained live cells green and dead cells red with no apparent difference. In order to assure all cells are stained, a 50:50 mixture of both kits was used for staining cocultured samples, hence the result is the viability of both cultures.

The viability of cocultured cells in week two was lower (65%) than for simple monocultures (Fig. 2C and D – MSC and *L. lactis* coculture). Note that bacteria-only was >80% and MSC-only was >90% (Fig. 2C and D). This is consistent with previous results that showed the viability of *L. lactis* in cocultures to decrease slightly with time [28]. Because the viability result of the coculture sample is a combination of two cell types, the 65% lower reading cannot be further resolved to either cell population.

### 3.3 Differentiation of MSCs in bacteria-laden microgels

**Fig. 3:**
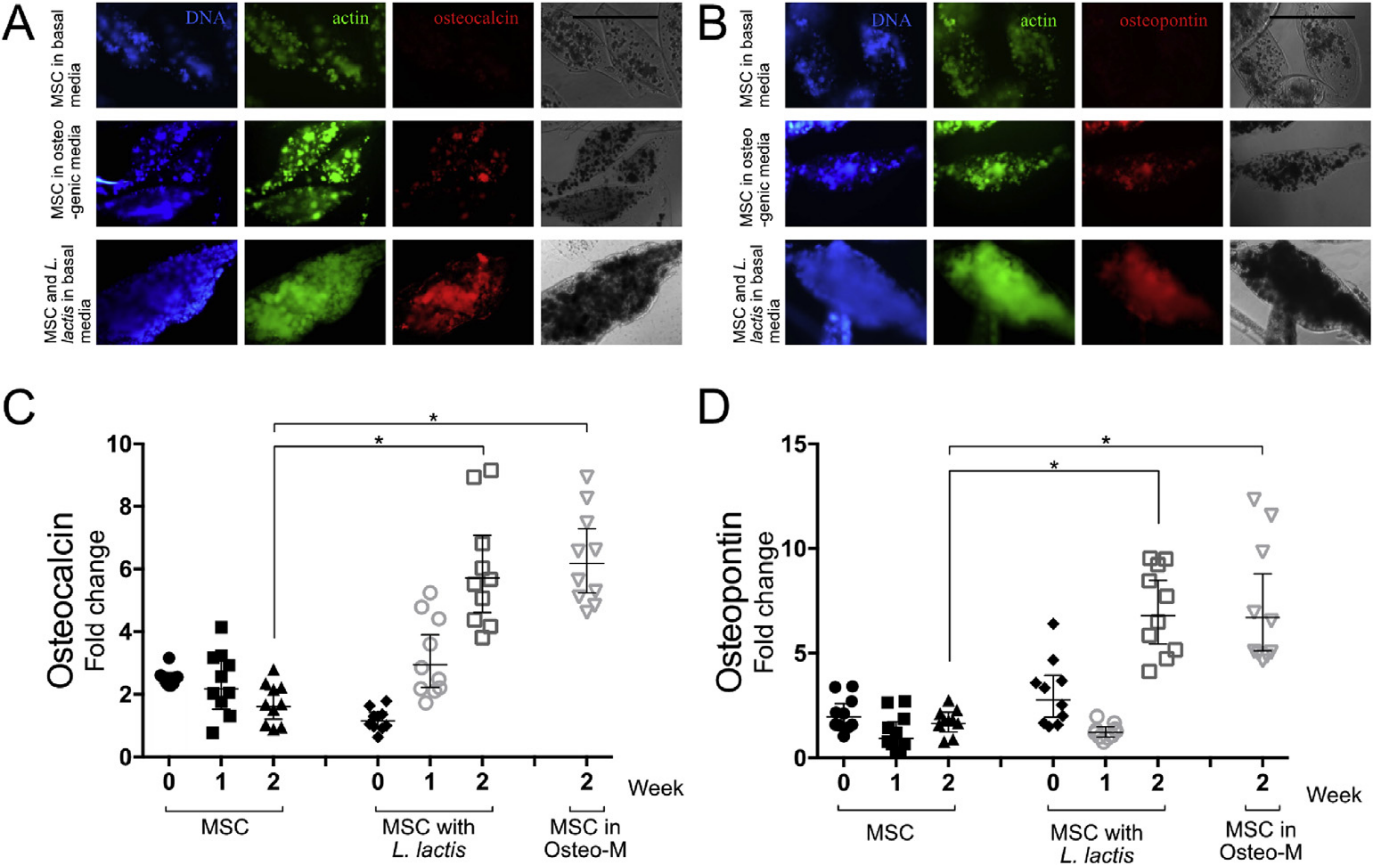
**Osteogenic differentiation of MSC via bacteria expressing fibronectin and BMP-2.** (A) hBM-MSCs were immunostained for osteocalcin (red), actin (green), and cell nuclei (blue). Cells were cultured in basal DMEM media with 2% FBS (top), with two *L. lactis* strains constitutively producing membrane-anchored FN and secreting BMP-2 in basal DMEM media with 2% FBS (bottom) and encapsulated in alginate hydrogel in osteogenic media (middle). Total integrated density corresponding to the red channel (osteocalcin) was quantified using ImageJ. Scale bar: 100 ​μm. (B) Samples from same experiment were also immunostained for osteopontin (red). (C) Graph shows osteocalcin area per microgel with standard deviation of the sample sets, N ​≥ ​10–20 microgels were analyzed for each condition. Integrated density corresponding to osteocalcin was found to be significantly higher in the bacteria-laden samples as well as samples cultured in osteogenic media after two weeks compared to MSCs without of bacteria. No detectable signal was found for 0- and 1-week old hydrogels. Data are presented as mean ​± ​SD and analyzed with ANOVA with a Tukey post hoc test. Significance levels are *p ​< ​0.05. (D) Graph showing similar trends to osteocalcin immunostaining was observed for osteopontin. MSCs, mesenchymal stem cells; DMEM, Dulbecco's Modified Eagle Medium; FBS, fetal bovine serum; FN, fibronectin; BMP-2, bone morphogenetic protein-2; ANOVA, analysis of variance; SD, standard deviation; hBM-MSCs, human bone marrow–derived mesenchymal stem cells.

To study the differentiation of MSCs within the bacteria-laden microgels, samples were immunostained for the bone-specific ECM proteins OPN ​as a marker for middle-stage differentiation and OCN ​as a marker for late-stage differentiation [39], [40]. As shown in Fig. 3, significant high expression levels were observed for OCN (Fig. 3A) and OPN (Fig. 3B) for samples of microgels containing MSCs and bacteria compared to microgels gels containing only MSCs (week 2). This demonstrates that microgels enable a commensalism symbiotic relationship between bacteria and MSCs to trigger cell differentiation in confined 3D microenvironments. Note that similar difference in expression of OPN and OCN was observed between week two samples of positive (MSCs in osteogenic media) and negative (in basal media) controls.

**Fig. 4:**
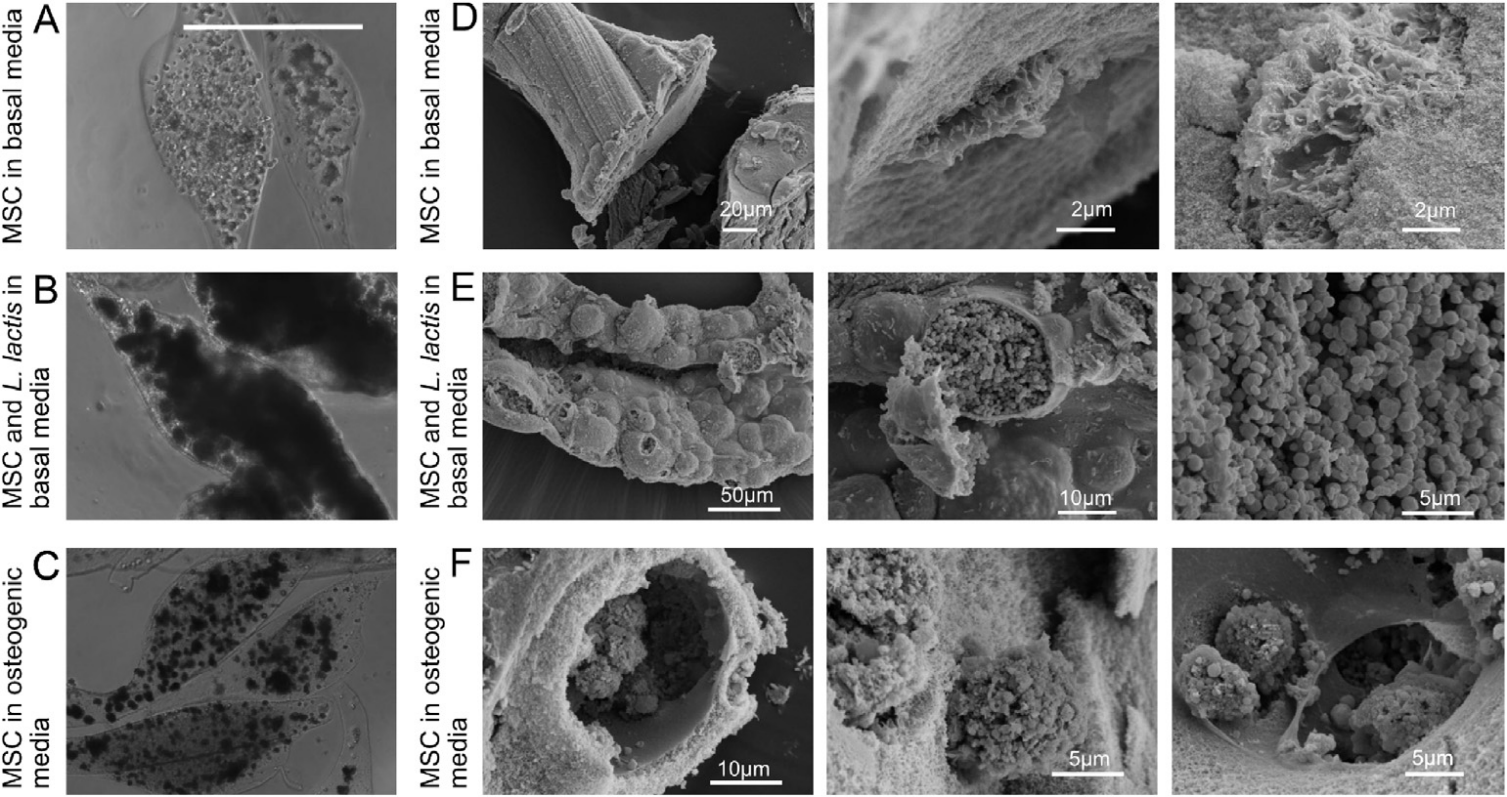
**SEM images of cell-laden alginate constructs.** Phase contrast images of alginate microgels with MSCs in basal media (A); alginate microgel with MSCs in osteogenic media (B); alginate microgel with MSCs containing two colonies of *L. lactis* (C) either expressing FNIII 7-10 or BMP-2 in a constitutive manner. Samples were fixed after two weeks of culture. Scale bar: 100 ​μm. SEM images of alginate construct with MSCs in basal media, the images show mark impressed by cell on the cross section of an alginate construct (D); alginate microgel with MSCs and two colonies of *L. lactis* overpopulating the space (E); alginate microgel with MSCs in osteogenic media, round entities covering the cells, cavities and fine membrane-like constructs (F). The hydrogels were slightly dehydrated/shrunken compared to their state in aqueous media (see also Fig. S2). MSCs, mesenchymal stem cells; SEM, scanning electron microscope.

The local distribution of bacteria and MSCs within the microgels was further investigated by SEM (Fig. 4). Whereas MSCs are distributed in relatively empty microgels, with different cell morphology in dependence of the use of basal or osteogenic medium, the coculture of both cell populations involves intimate contact between eukaryotic and prokaryotic cells. This result confirms the symbiotic relationship between both cell population in confined spaces allowed by the microgels.

**Fig. 5:**
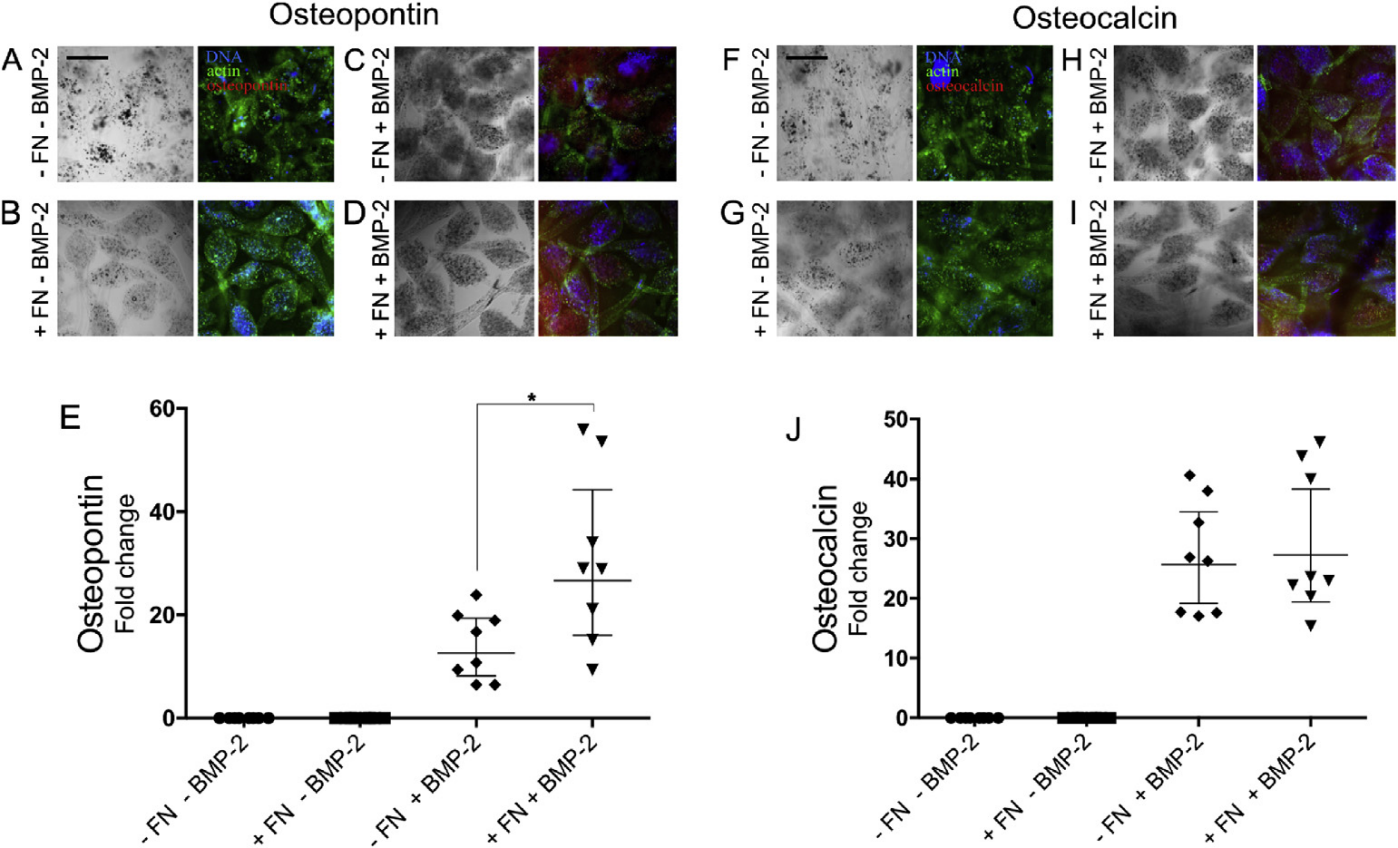
**Osteogenic differentiation of MSCs using both populations of bacterial expressing fibronectin and BMP-2** as observed by the expression of Osteopontin and Osteocalcin. Phase contrast and fluorescent images of (A, F) MSCs encapsulated in alginate hydrogel with no bacteria. (B, G) MSCs with *L. Lactis* containing inducible expression vector for producing membrane-anchored FN. (C, H) MSCs with *L. Lactis* containing inducible expression vector for secreting BMP-2. (D, I) MSCs with both *L. lactis* strains producing membrane-anchored FN and secreting BMP-2. Hydrogels were stained after two weeks for osteopontin (red), actin is (green), DNA (blue). Scale bar: 100 μm. (E) Graph showing the production of osteogenic markers osteopontin (OPN) was significantly higher in MSCs with bacteria producing both FN and BMP-2 compare to BMP-2 only. No detectable signal was observed for FN only and control (no FN and no BMP-2). (J) Similar graph for osteocalcin (OCN) shows detectable signal for samples containing bacteria which produces BMP-2. However, no statistically significant difference in the OCN level was observed between samples containing MSCs in with bacteria producing both FN and BMP-2 compare to BMP-2 only. No detectable signal was observed for FN only and control (no FN and no BMP-2). Graph shows osteopontin/osteocalcin area per microgel with standard deviation of the sample sets, N ≥ 8–16 microgels were analyzed for each condition. Data presented as mean ± SD and analyzed with ANOVA with a Tukey post hoc test. Significance levels are ∗p < 0.05. MSCs, mesenchymal stem cells; FN, fibronectin; BMP-2, bone morphogenetic protein-2; ANOVA, analysis of variance; SD, standard deviation.

The synergistic signaling between growth factors and integrins binding regions for enhancing stem cell differentiation has been extensively studied in recent years [41], [42]. In particular FN and its fragments for engineering synergistic BMP-2 microenvironment for osteogenic differentiation has been well documented [27]. In this experiment, we explored the feasibility of our microgels—that maintain both population of cells in intimate contact—to trigger the synergistic effect of both populations of bacteria expressing FN and BMP-2 by encapsulating MSCs with one of the two inducible *L. lactis* colonies for either expressing FNIII 7-10 fragment or BMP-2. We used inducible expression vectors for the same two genes: BMP-2 and FNIII 7-10. *L. lactis* were induced daily by 10 ​ng/mL nisin which has previously shown to sustain 200 ​ng/mL secretion of BMP-2 over a 24 ​h period [28]. The concentration of BMP-2 is maintained locally within the microgel. As shown in Fig. 5, the OPN and OCN marker are more expressed in alginate construct containing either BMP-2 or BMP-2 together with FNIII 7-10 compared to FNIII 7-10 only or encapsulated MSCs without bacteria. The coculture containing both BMP-2 and FNIII 7-10 shows significant increase in OPN as compared to BMP-2 alone. The higher yet not significant readout for OCN was also observed in the same comparison, hence further reaffirming the enhanced osteogenic differentiation through the synergistic effect of both expressed proteins in our micron-sized 3D hydrogel.

### 3.4 Pearl lace 3D printing

**Fig. 6:**
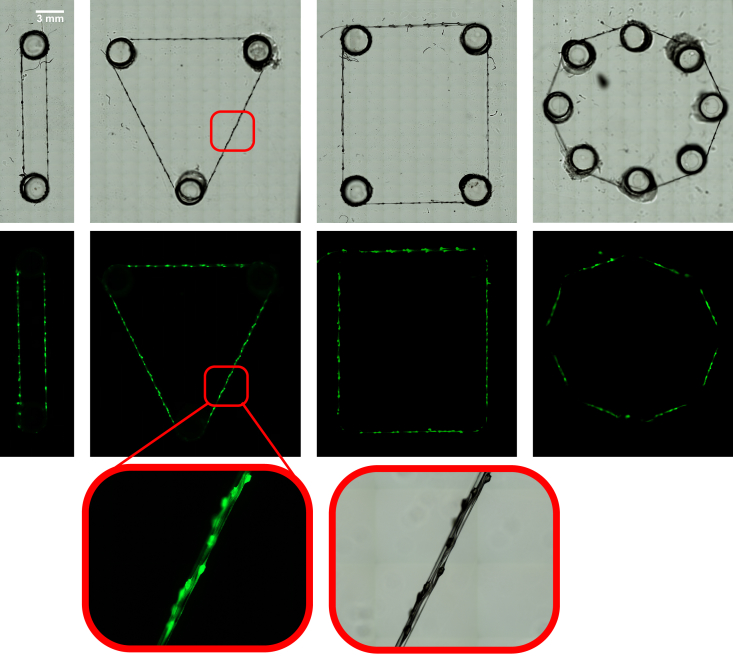
**Bright-field and fluorescent images of pearl lace 3D printed simple geometries.** The 3 ​mm in diameter circular discs were laser cut through 3.00 ​mm acrylic (PMMA) sheet and glued using epoxy on a second 3.0 ​mm acrylic sheet. One-micron-sized green (450/480) fluorescent polystyrene microspheres were encapsulated within alginate pearl lace construct. The 90-degree cross-sectional thickness varies from 10 μm at an interlinking unit between two adjacent pearls to 50 μm across a pearl. There are four rounds of thread in the triangle geometry. The images were taken as a scan over a 30 ​× ​30 mm square (Supplementary Fig. S3) using EVOS Fl Colour Cell Imaging System at 10X objective and stitched together using EVOS owns software.

The unique interlinking pearl lace microstructure explored in this study was used to create simple geometrical shapes (line, triangle, square, and circle; Fig. 6 and S3). To demonstrate the encapsulation efficiency and the imaging of the transparent alginate-based microgel, one-micron-sized fluorescent beads were encapsulated inside the construct used for making geometrical shapes (Fig. 6). The thickness of the structure ranges from 10 μm at the interlinking unit between two adjacent pearls to 50 μm at a pearl unit. Supplementary Video 5 demonstrates the robustness of the thread made from this very thin microgel ​as it wraps around a coverside.

Supplementary video related to this article can be found at https://doi.org/10.1016/j.mtbio.2019.100011

The following is the supplementary data related to this article:

Supplementary video 5 5 Supplementary video 5

## 4 Conclusion

Cell-laden micron-sized hydrogels as miniaturized natural microenvironments can be used for studying cellular behavior such as migration, survival, proliferation, and differentiation in vitro [10], [43]. Droplet microfluidics technology is ideally suited for the construction of cell-laden microgels with micron size distributions. An inherent quality of microfluidic systems is its reliability of producing monodisperse constructs which enables quantitative studies to be performed using them. This feature in addition to their high data throughput give them a great potential to contribute to fundamental biological studies, pharmacological screenings, and personalized medicine [44]. In this study, we produced monodisperse alginate-based hydrogels encapsulating different population of prokaryotic and eukaryotic cells using one-step microfluidic fabrication. Benefiting from the fast gelation kinetics of alginic acid with calcium, different hydrogel constructs were made. Microfluidic parameters can be adjusted to have microconstructs in the form of individual beads or linked together via a thread made of a same material. The so-called linked beads in a shape of pearl lace structures enables both the individual compartmentalization as seen in separate capsules as well as being linked to other identical units via a highly controllable link made from same material.

The connectivity of pearl lace hydrogels can provide a way of gradient studies in which the population of each cell type and so its relative density can be controlled. It can also be utilized for time-series indexing studies as well as providing a mean for a low-cost, easy to fabricate 3D bioprinting prototypes as demonstrated in this study.

Also because of their relatively small dimension, a few micrometers thickness, they are readily compatible with standard microscopy techniques without needing to be sectioned [6]. Sizes in microscale also offer efficient material-to-cell volume ratios with improved nutrients by diffusion over distances that are often on the order of millimeters [2]. This platform and the unique interlinking pearl lace microstructure has the potential to form larger tissue constructs with homogeneous material properties through cell-containing micromodules.

To conclude, we have engineered a microfluidic system for high encapsulation efficiency and cell viability by recreating bacteria-based materials [28] into a miniature scale. MSC differentiation is triggered by bacteria cohabiting in the same compartmentalized microconstruct. The cohabitation of both eukaryotic and prokaryotic cells in controllable microenvironments has been explored as an in vitro examination model with no in vivo prospect due to the potential potent immune response in host to the presence of bacteria. Even though once cells are differentiated, bacteria can be killed through a build-in kill switch [45] or antibiotic combination such as penicillin and streptomycin since bacteria does not have resistance genes for either ones. Microgel in this study has been utilized as a proof of concept for modeling a tuneable platform in which both hydrogel acting as an ECM as well as production and release of growth factor can be both engineered at a low cost with high precision spatiotemporal control. It has been an attempt to further engineer more aspects of an in vitro system, paving the way for study of cells and their interaction with adjustable dynamic ECM-like environment with greater control. This is a versatile encapsulation platform which enables coculture and concentration-dependent studies to be carried out in a unique microcontrolled environment for MSC engineering. In this particular model, it can also be utilized as a drug-screening platform once the chemical profile of chemicals produced by bacteria is identified and measured using spectroscopy against profile obtained from drug(s) of interest.

## Declaration of interests

The authors declare that they have no known competing financial interests or personal relationships that could have appeared to influence the work reported in this paper.

## Funding

The support from the UK Engineering and Physical Sciences Research Council (EP/P001114/1) is acknowledged.
