# Supplementary material for: Bacteria-laden microgels as autonomous three-dimensional environments for stem cell engineering
Source: Mater Today Bio. 2019 Jun 18;2:100011. doi: 10.1016/j.mtbio.2019.100011 (PMC7061548; doi:10.1016/j.mtbio.2019.100011)
Supplement: Multimedia component 6 [file mmc6.docx]

**Appendix A. Supplementary data**

**Bacteria laden microgels as autonomous 3D environments for stem cell engineering**

*Kimia Witte, Aleixandre Rodrigo-Navarro, Manuel Salmeron-Sanchez**

Kimia Witte, Aleixandre Rodrigo-Navarro, Manuel Salmeron-Sanchez

Center for the Cellular Microenvironment, University of Glasgow, G12 8LT, UK

E-mail: [manuel.salmeron-sanchez@glasgow.ac.uk](mailto:manuel.salmeron-sanchez@glasgow.ac.uk)

**Figure S1. Bacteria laden unlinked and linked pearl-lace alginate constructs. (**A) Brightfield of individual beads were made at relatively low flow rates (100 - 200 μL h^-1^ for water phase and 1000 – 2000 μL h^-1^ for oil phase) as compared to those of (B) linked pearl-lace microgels (300 – 1000 μL h^-1^ for water and 3000 – 10000 μL h^-1^ for oil). (C) Images of encapsulated bacteria taken at day 1, 2 and 3. Bacteria’s growth was not suppressed with antibiotics. Scale bar: 100 μm.

**Figure S2. SEM and TEM images of cell-laden alginate constructs.** (A) Images of alginate network from a sliced empty microgel. (B) TEM image of a whole MSC in alginate construct (left); MSC cilia interaction with alginate network (middle); *L. lactis* colonies (right). The hydrogels were slightly dehydrated/shrunken compared to their state in aqueous media in both SEM and TEM images.

**Figure S3. Images of pearl-lace 3D printed simple geometries.** Tope left image: The 3mm in diameter circular discs were laser-cut through 3.00 mm acrylic (PMMA) sheet and glued using epoxy on a second 3.0 mm acrylic sheet. Top right and bottom left image: printed construct around the discs. Bottom right image: printed construct around a cover slide (for video of printing around cover slide see supplementary video 5).

**Please refer to five separately submitted files for the supplementary videos.**
